# One-stage VATS surgery for synchronous bilateral lung lesion: a safe and feasible procedure

**DOI:** 10.1186/s13019-023-02215-3

**Published:** 2023-04-11

**Authors:** Zhe Wang, Xue Zhang, Xusheng Sun, Junfeng Liu

**Affiliations:** 1 Department of Thoracic Surgery, Hebei Medical University Fourth Hospital, 12 Jiankang Road, Shijiazhuang, 050011 Hebei Province China; 2 Medical Oncology, Hebei Medical University Fourth Hospital, Shijiazhuang, China

**Keywords:** VATS, Lung, Complications

## Abstract

**Background:**

Synchronous bilateral lung lesions are emerging as a common but tricky disease for surgical management. Whether one or two-stage surgery should be taken remains in debate. We retrospectively analysed 151 patients who underwent one and two-stage Video Assisted Thoracic Surgery (VATS) to investigate the safety and feasibility of the two surgical approaches.

**Methods:**

A total of 151 patients were included in the study. Propensity score matching was performed to minimize the baseline characteristics difference between one and two-stage groups. Clinical factors including in-hospital days after surgery, chest tube drainage days, types and severity of post-operative complications were compared between the two groups. Logistic univariate and multivariate analyses were used to find the risk factors for post-operative complications. Nomogram was built to select the low risk candidates for the one-stage VATS.

**Results:**

After propensity score matching, 36 one-stage and 23 two-stage patients were enrolled. The age (*p* = 0.669), gender (*p* = 0.3655), smoking status (*p* = 0.5555), pre-operative comorbidity (*p* = 0.8162), surgical resection (*p* = 0.798) and lymph node dissection (*p* = 9036) were balanced between the two groups. There was no difference in post-surgery hospital days (8.67 ± 2.68 versus 8.46 ± 2.92, *p* = 0.7711) and chest tube retaining days (5.47 ± 2.20 versus 5.46 ± 1.95, *p* = 0.9772). Moreover, post-operative complications also showed no difference between one-stage and two-stage groups (*p* = 0.3627). Univariate and multivariate analysis revealed that advanced age (*p* = 0.0495), pre-surgical low haemoglobin (*p* = 0.045) and blood loss (*p* = 0.002) were risk factors for post-operative complications. Nomogram built with the three risk factors showed reasonable predictive value.

**Conclusions:**

One-stage VATS for synchronous bilateral lung lesion patients was proved to be a safety procedure. Advanced age, pre-surgical low haemoglobin and blood loss may predict complications after surgery.

## Background

Low dose computed tomography (LDCT) is widely used in lung cancer screening. As a result, an increasing number of small nodules that appear as ground-glass opacities and solid nodules are found [1]. There has been numerous research on the diagnosis and treatment of solitary lung lesions, but very few on synchronous bilateral lung lesions (SBLL). SBLL patients, however, are not rarely seen. It is often confusing for the surgeons to make clinical decision on whether these patients should receive surgical treatment. As there is no consensus on the surgical management of bilateral lung lesions, the treatment often varies among surgeons.

In recent decades, video-assisted thoracic surgery (VATS) has become the standard of care for patients with pulmonary nodules. With the maturation of VATS technique in solitary nodules surgery, some surgeons are applying it in SBLL patients. However, considering the risk of post-operative pulmonary dysfunction and complications, one-stage bilateral VATS lung resection is not used routinely [2]. Some surgeons made exploratory investigation on the one-stage VATS and achieved inspiring results [3, 4]. In this study, we retrospectively reviewed perioperative data of 151 patients who underwent VATS for SBLL in our centre and compared the outcomes after propensity score matching (PSM). Furthermore, we also investigated the potential risk factors for post-operative complications in one-stage surgery. To our knowledge, this is the first study that demonstrate the safety and feasibility of one-stage VATS for SBLL treatment.

## Methods

Medical records of lung surgeries performed at Hebei Medical University Fourth Hospital between December 2014 and December 2020 were collected. To protect the privacy of the patients, their names and IDs were desensitized. During this period, a total of 10,711 patients underwent lung resections, with 109 synchronous bilateral lung lesions (SBLL) receiving one-stage bilateral VATS lung resection. Meanwhile, two-stage VATS surgery was performed on 42 SBLL patients. Medical data including sex, age, pre-operation comorbidity, laboratory test, pulmonary function test, surgery type, lymph node dissection, chest tube duration, post-operative days and complications were all collected. Complications after surgery were defined according to the Clavien–Dindo classification [5].

### Surgery procedures

General anaesthesia was implemented with intravenous remifentanil and inhalational sevoflurane. Double-lumen tracheal intubation or single-lumen intubation with bronchus blocker was used to maintain the contralateral ventilation to the surgery side. Surgeries were performed by experienced surgeons who routinely perform VATS surgery for over 5 to 10 years. The one- or two-stage VATS were at the choice of the surgeon, according to the patient physical and psychological condition, performance status, tumour size and planned resection scope, etc. For younger, better performance status patients, some surgeons tended to resect all the lesions at one time. Whereas, for some older patients, if the lesions were small and located peripherally, wedge resections were suitable, and one-stage bilateral VATS could be chose by the surgeons. Some cases showed typical invasive adenocarcinoma which requires lobectomy at one side, and at the other side the lesion may need complex segmentectomy. A two-stage VATS surgery may be selected by most surgeons. Uni-portal, bi-portal or tri-portal VATS approaches were decided by the surgeon’s preference. Normally, in one-stage bilateral surgery, wedge resection and frozen biopsy were performed for the major lesion located at the peripheral of the lung. Anatomical segmentectomy or wedge resection was performed for small lesions less than 2 cm in diameter, depending on their location: central or peripheral. Lobectomy was performed for lesions larger than 2 cm in diameter or pathologically reported as adenocarcinoma in the frozen biopsy. Two-stage surgery was defined as that a planned second resection was performed within one year from the first surgery. In two-stage surgery, the major lesion was resected in the first stage, which normally needed lobectomy. In the second stage, the resection plan was determined by the lesion characteristics and pulmonary function. Chest tubes were routinely placed on each surgical side. Among all the subjects, one case was converted to thoracotomy intraoperatively due to difficult lymph node dissection.

### Postoperative management

Antibiotics and analgesics were prescribed routinely. Chest radiography and laboratory test were issued within 2 days after surgery. Early ambulation and coughing were encouraged. Enteral nutrition was conducted on the first day after surgery. The chest tube was removed when there was no sign of air leak in X-ray and drainage was less than 200mL per day.

### Statistical analysis

Continuous variables were presented as mean values ± standard deviation. Categorical variables were presented as numbers. Continuous variables with normal distribution were compared using two sample t test. Categorical variables were compared by chi-square test. For the patients who were planned to have bilateral lung surgery, surgeons tended to choose younger ones for the one-stage surgery. To resolve this potential bias, propensity score matching was introduced to balance the baseline characteristics between one- and two-stage group. While the analysis for the one-stage group complications was performed in the pre-matched “whole” data, as there was no comparation between one- and two-stage group. Logistic regression was performed to identify the risk factors of post-operative complications in one-stage surgery. Chi square test and Mann-Whitney-U test was performed to investigate the difference in number and grade of the complications between the two groups. The receiver operating characteristics (ROC) curves were plotted for the risk factors of one-stage VATS complications A nomogram was established to predict the risk of complications of one-stage VATS. Harrell Consistency Index (C-Index) and area under ROC curve (AUC) were used to evaluate the nomogram, and calibration curve were used to validate the nomogram performance. Statistical analysis was performed with R (version 4.2.0) in R Studio software (Version 1.4.1717, Boston, MA). For propensity score matching analysis, package “MatchIt” was used. Significance level was set at 5%.

## Results

### Patient characteristics

A total of 109 patients underwent one-stage bilateral VATS lung resection, while 42 received two-stage treatment during this time. For the two-stage patients, values of “post-surgery days” and “chest tube days” were the average of the two operations. Systematic lymph node dissection was marked if it was performed on either side of the mediastinal during the operations. Lymph node sampling was recorded when it was performed on both sides, or only one side but without systematic lymph node dissection on the other side. The baseline of patient characteristics was shown in Table 1.

**Table 1:** Patient characteristics before and after propensity score matching

Characteristics	Pre-match			Post-match		
	One-stage	Two-stage	*p* value	One-stage	Two-stage	*p* value
Gender			0.9118			0.3655
Female	56	22		16	13	
Male	53	20		20	10	
Age	57.85 ± 8.21	55.55 ± 14.25	0.2169	56.42 ± 9.35	54.78 ± 16.55	0.669
Smoke*			0.5568			0.5555
Yes	33	10		12	6	
No	76	32		24	17	
Pulmonary function***						
FEV1 (L)	2.64 ± 0.57	2.54 ± 0.58	0.3601			
FEV1/FVC	76.96% ± 4.87%	75.13% ± 5.37%	0.0849			
Comorbidity			0.0257			0.8162
Yes	37	23		12	7	
No	72	19		24	16	
Post-surgery days	8.72 ± 3.36	8.4 ± 2.64	0.5765	8.67 ± 2.68	8.46 ± 2.92	0.7711
Chest tube days	5.64 ± 2.42	5.56 ± 2.05	0.8452	5.47 ± 2.20	5.46 ± 1.95	0.9772
Surgery			0.001106			0.798**
L + L	4	7		3	3	
SL + L	43	23		17	9	
SL + SL	62	12		16	11	
Lymph node dissection			0.07427			0.9036*
Systematic	67	34		27	16	
Sampling	11	2		3	2	
None	31	6		6	25	
Pathology of lesions			0.021			
Pre-cancerous	7	8				
Cancer	160	62				
Benign	79	20				
Post-operative complications			0.8669			0.3627
Yes	33	14		10	14	
No	76	28		26	9	
Complication type			–			
Hydrothorax (thoracentesis)	15	6		4	1	0.3635*
Bronchoscopic sputum suction	3	2		3	2	
Other	14	6		3	6	

Comorbidity: including diabetes, heart disease, hypertension and other diseases before surgery

*L* Lobectomy, *SL*  Sub-lobectomy, *FEV1* Forced expiratory volume for the first second, *FVC* Forced vital capacity

*Past smokers are categorized as ‘yes’ in smoking status, as many patients claimed to be past smokers actually quit smoking on the day when diagnosed

**Fisher exact test

***In two-stage surgery, pulmonary function data are results before the first surgery

Although the gender, age, and smoking status were not statistically different before propensity matching (PSM), the percentage of smokers in one-stage surgery was higher than that in two-stage group. Moreover, there were significantly more patients with comorbidities such as hypertension, coronary artery disease, and diabetes in two-stage group, which could possibly be the reason for their two-stage operations. Some variables such as neutrophils/lymphocyte ratio (NLR), platelet (PLT), haemoglobin (HB), surgery duration, and blood loss were not included into the PSM, because the first operation had a remarkable influence on these factors, making them pointless to compare.

### Overall surgery results

Most patients recovered quickly without any complications. Bedside chest x-ray was routinely taken on the first or the second post-operative day and 86.1% of patients showed well-inflated lungs with clear thoracic cavities (94 in 109 one-stage and 36 in 42 two-stage). The average chest tube removal days were 5.64 in one-stage and 5.56 in two-stage. The average discharge days were 8.72 in one-stage and 8.4 in two-stage. (Table 1)

### Propensity score matching and outcomes

**Fig. 1:**
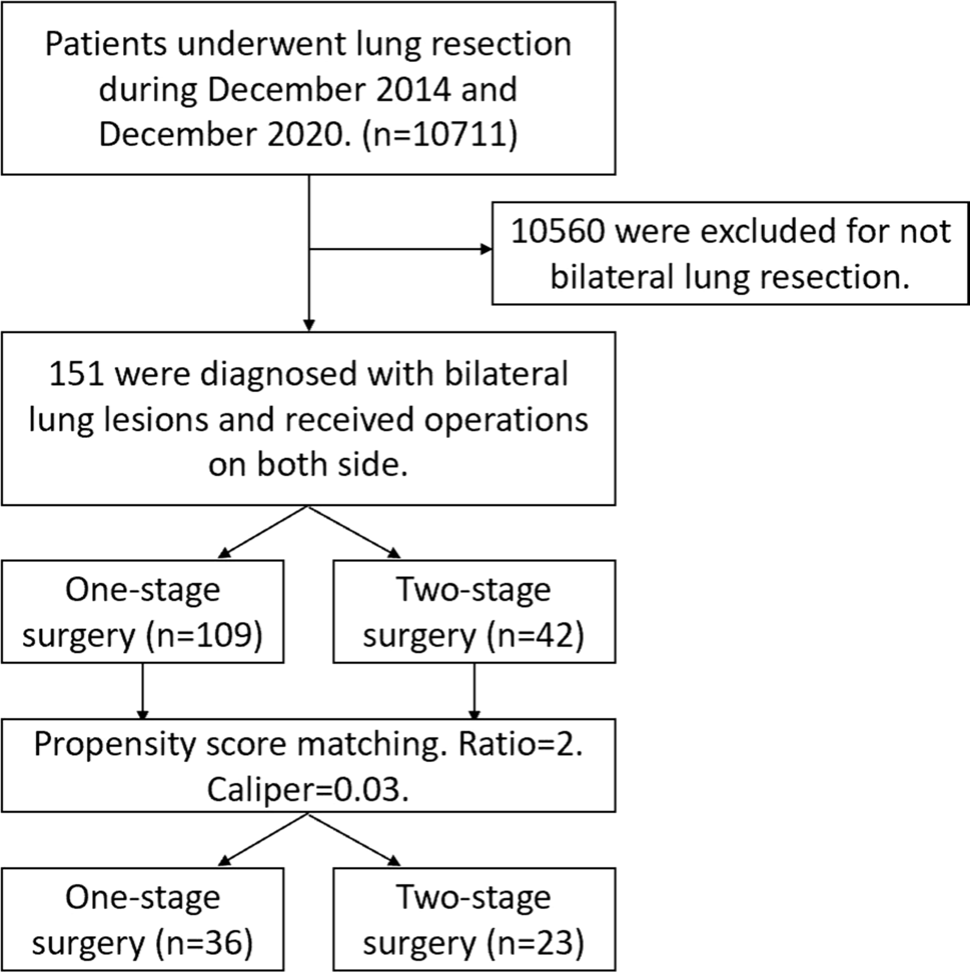
Flowchart of propensity score matching

**Fig. 2:**
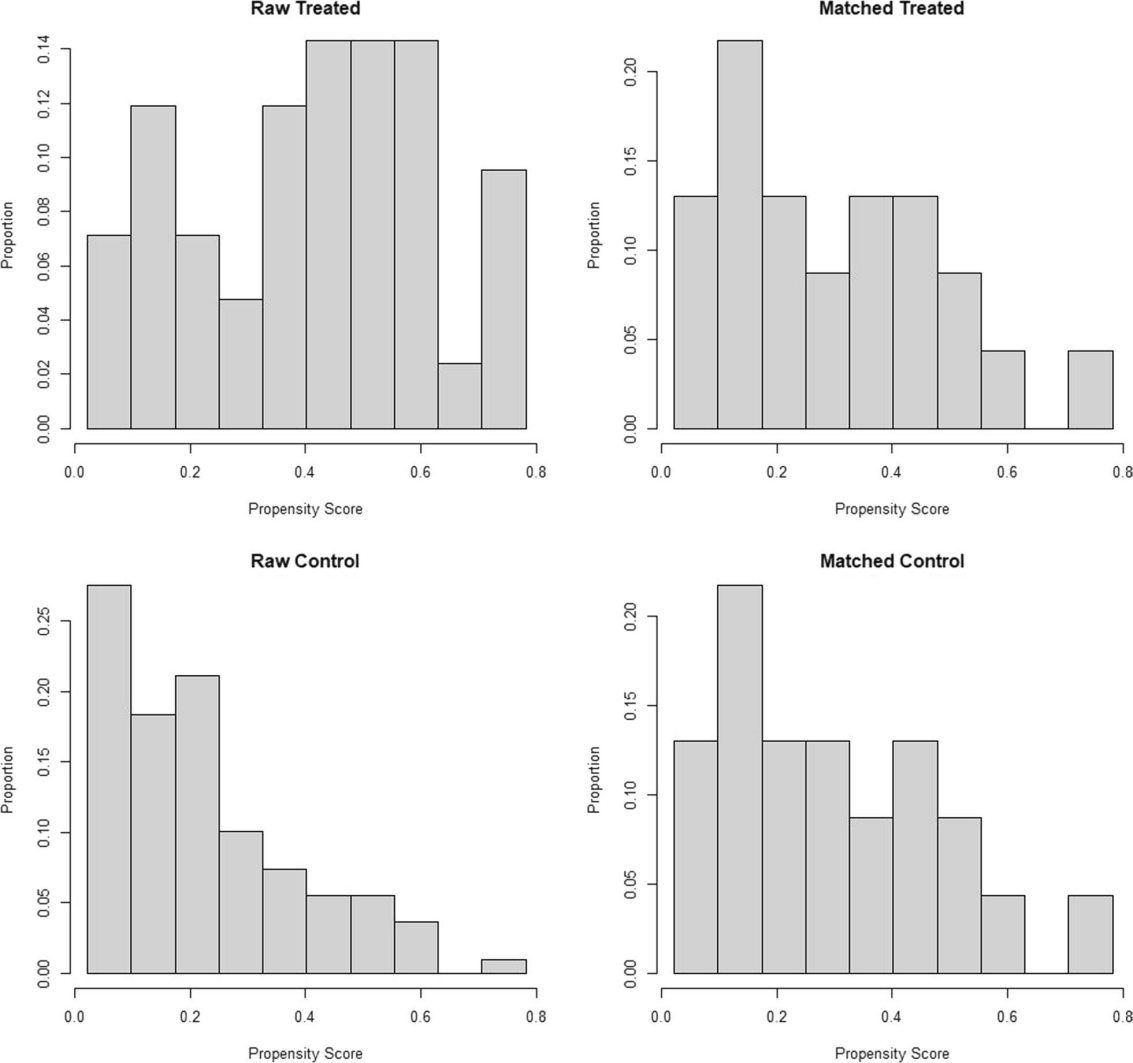
Propensity score before and after matching. Treated and control group were not matched in raw data, but well matched after propensity score matching

To minimize the difference of patients’ characteristics between two groups, propensity score matching (PSM) was adopted. The process was summarized in Fig. 1 as mentioned above. PSM was conducted using “MatchIt” package with nearest-neighbour method and a caliper of 0.03. Figure 2 showed the propensity score matching of raw data and matched data. After matching, the control and treated groups achieved well-balanced results. Finally, 36 one-stage patients and 23 two-stage patients were included in the analysis set.

In the post-match set, there was no significant difference in post-operative hospital days (8.67 ± 2.68 versus 8.46 ± 2.92, *p* = 0.7711) and chest tube retaining days (5.47 ± 2.20 versus 5.46 ± 1.95, *p* = 0.9772). More importantly, post-operative complications also showed no difference in number of cases between one-stage and two-stage groups, statistically (*p* = 0.3627). Further analysis according to the Clavien–Dindo classification was performed. The grade of the complications between the two groups also showed no significant difference (*p* = 0.3961) (Table 2). No perioperative death occurred.

**Table 2:** The Clavien–Dindo Classification for post-operative complications

The Clavien–Dindo classification	One-stage	Two-stage	*p* value*
I	3	2	0.3961
II	7	2	
IIIA	6	4	
IIIB	0	0	
IVA	1	0	

*Mann–Whitney U test

### Risk factors of post-operative complications in one-stage surgery

**Fig. 3:**
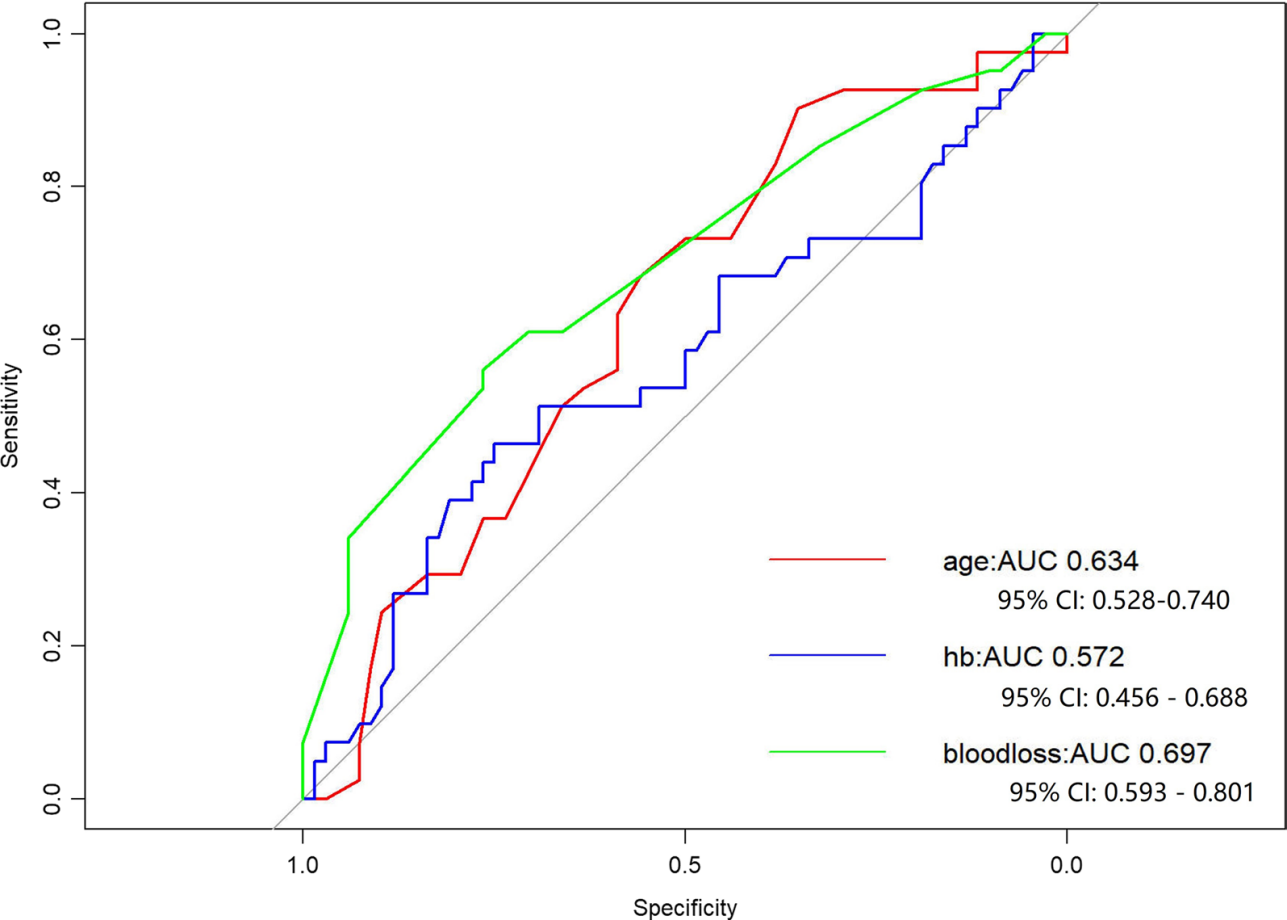
ROC curve for the risk factors of post-operative complication in one-stage surgery

Results showed that the complications of one-stage surgery were no more common and severe than that of two-stage surgery. Hence, one-stage VATS may be an optimal choice for SBLL patients. However, lung resection was still a major procedure with potential complications, and some may even lead to life threatening consequence. Therefore, risk factor analysis was subsequently performed on the all 151 one-stage patients to reveal those which may possibly be correlated to adverse conditions. As shown in Table 3, univariate analysis showed that age, HB, surgery duration, and blood loss may be related to post-operative complications in one-stage surgery (*p* < 0.05). Furthermore, in multivariate analysis, results demonstrated that advanced age (*p* = 0.04951, OR 1.07, 95% CI 1–1.15), pre-surgical low HB (*p* = 0.045, OR 1.04, 95% CI 1–1.08) and increased blood loss (*p* = 0.002, OR 1.02, 95% CI 1.01–1.04) were risk factors for complications in one-stage surgery. Receiver operating characteristics curves were plotted for the risk factors. (Fig. 3). Area under the ROC curve and its 95% CI were also calculated.

**Table 3:** Risk factors for post-operative complications of one-stage surgery

Characteristics	One-stage		*p* value*		
	With complications	Without complications	Univariate	Multivariate	
Gender			0.101617		
Female	13	43			
Male	20	33			
Age (mean ± SD)	62 ± 6.67 (46–70)	56.55 ± 8.52 (36–72)	0.01427		0.04951 (OR 1.07 95% CI 1–1.15)
Smoke			0.3682		
Yes	8	25			
No	25	51			
Comorbidity			0.429481		
Yes	13	24			
No	20	52			
Surgery					
L + L	2	2	1		
SL + L	13	30	0.427		
SL + SL	18	44	0.389		
Lymph node dissection					
Systematic	20	47	1		
Sampling	5	6	0.30982		
None	8	23	0.68049		
NLR	2.659 ± 1.423	2.656 ± 1.242	0.9904		
PLT	233 ± 49.31	247 ± 64.85	0.255		
HB	133.83 ± 14.54	127.26 ± 13.75	0.0295		0.045 (OR 1.04, 95% CI 1–1.08)
Surgery duration	228.76 ± 86.55	186.82 ± 69.50	0.011287		0.629
Blood loss	101.82 ± 46.26	61.78 ± 33.96	< 0.001 (3.53e−05)		0.002 (OR 1.02, 95% CI 1.01–1.04)

*NLR* Neutrophils/lymphocyte ratio, *PLT* Platelet, *HB* Haemoglobin

*Logistic regression was performed to calculate the univariate and multivariate *p* values. Potential risk factors with *p* values less or nearly 0.05 were included into further multivariate analysis

### Nomogram and its evaluation

**Fig. 4:**
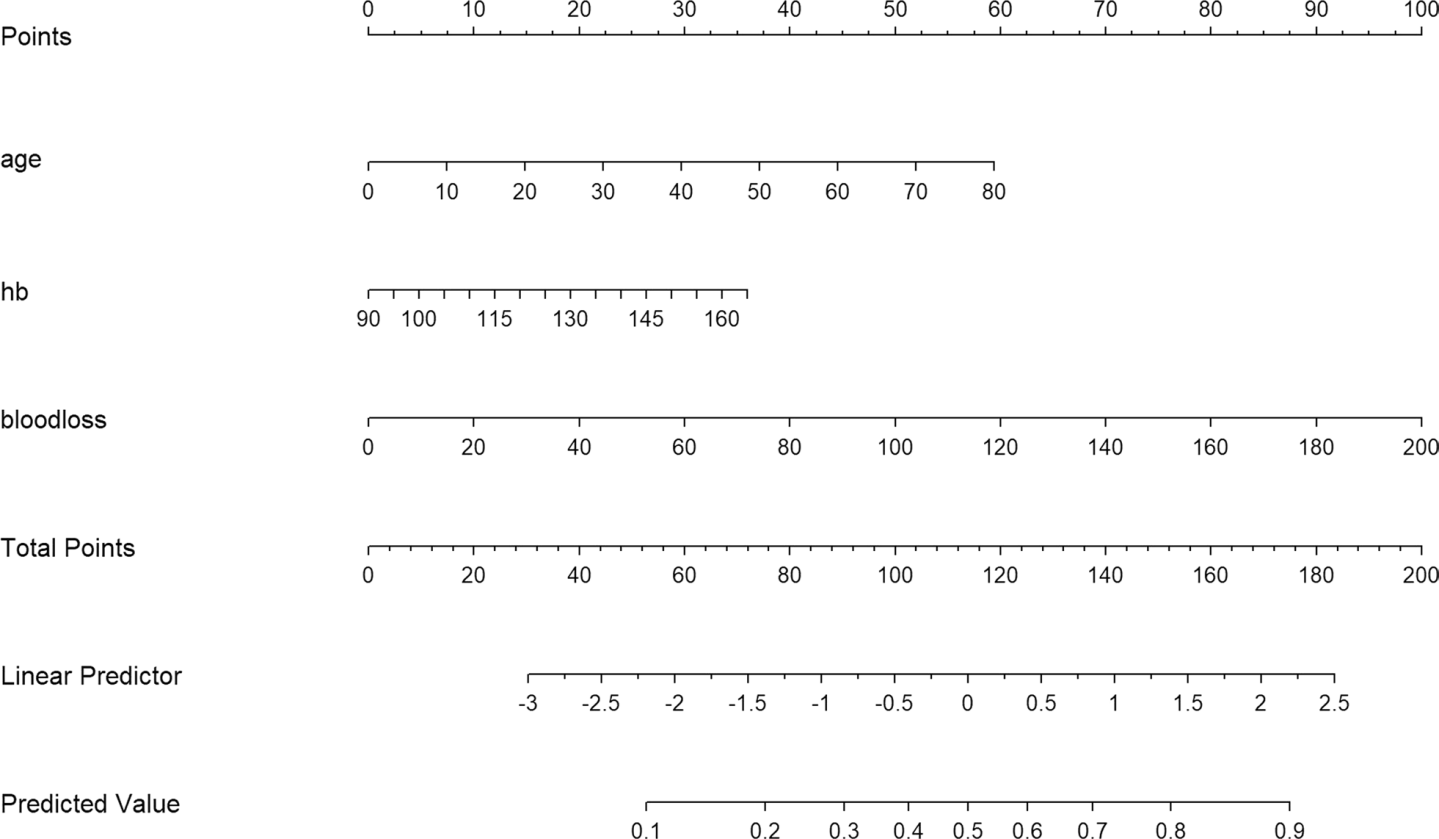
Nomogram for the prediction of post-operative complication in one-stage surgery

**Fig. 5:**
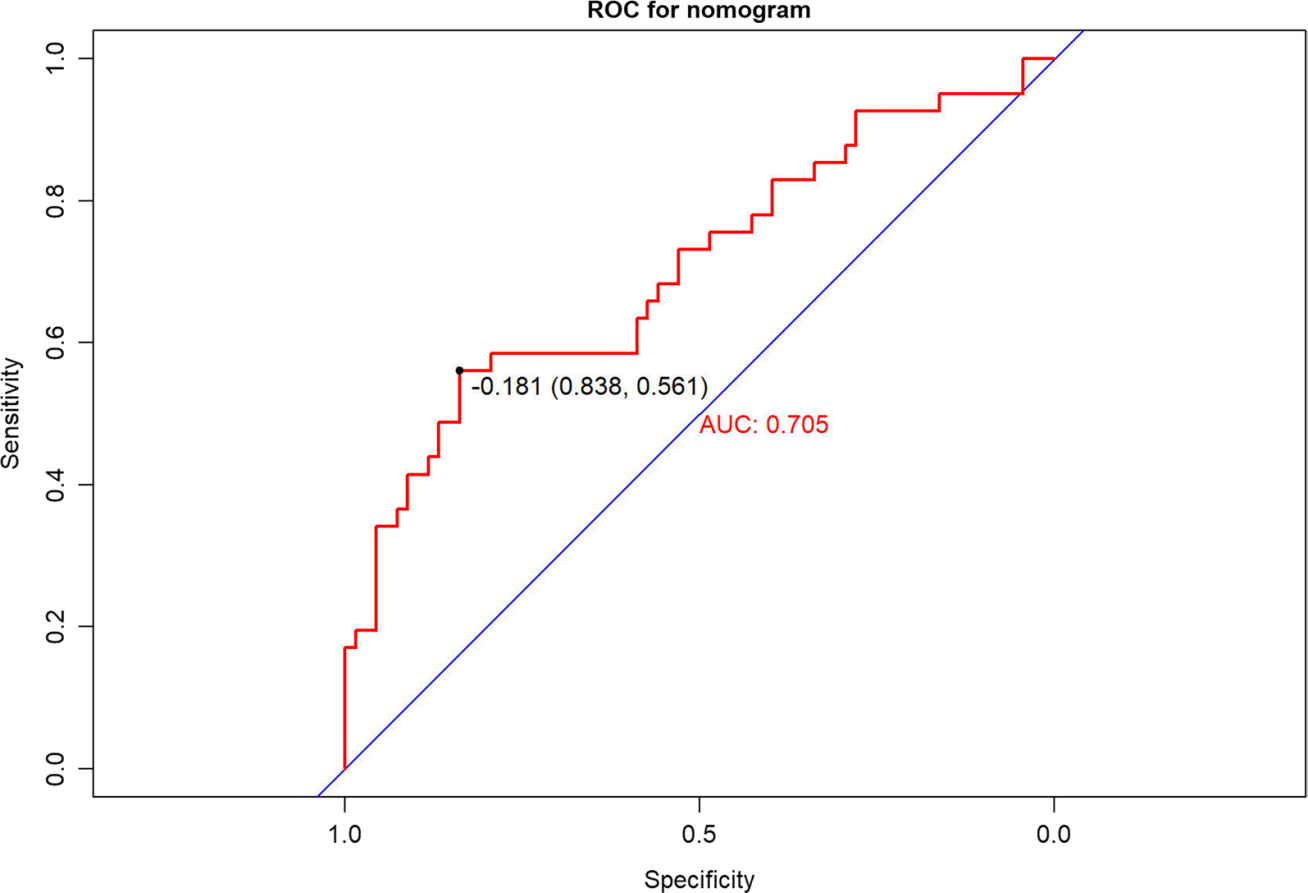
ROC curve for the nomogram

**Fig. 6:**
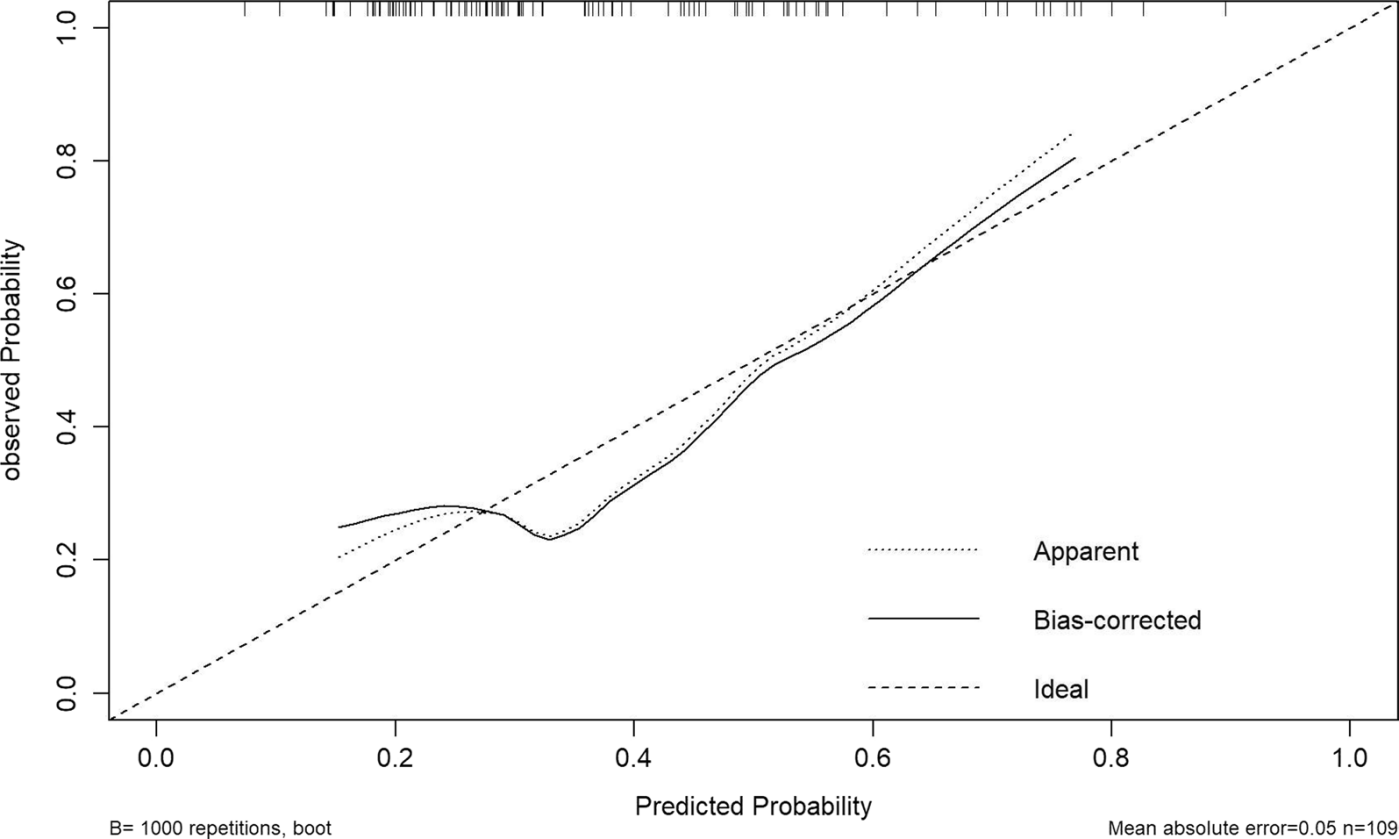
Calibration curve for the nomogram

We combined the risk factors from multivariate analysis, age, HB and blood loss, into the building of the nomogram for the prediction of one-stage surgery complications (Fig. 4). The line on the bottom indicates the probability that complications may occur after one-stage surgery. Area under the ROC curve of the nomogram was 0.705 (95% CI 0.600–0.811) in the model from the one-stage surgery data, with a sensitivity of 83.8% and a specificity of 56.1% (Fig. 5). The calibration curve for the nomogram demonstrated reasonable consistency between actual and predicted results (Fig. 6). These findings indicated that the nomogram could provide suggestions for the risk of one-stage surgery complications.

## Discussion

Choosing a proper surgical plan for synchronized bilateral lung lesion patients has been emerging as a critical problem for thoracic surgeons. Moreover, there is still no consensus on whether one-stage or two-stage surgery should be adopted. Originally, bilateral pulmonary resection was performed mostly on a two-stage basis in our centre. As the VATS technique developed, one-stage surgery cases were also growing. In this study, we retrospectively reviewed 151 cases of such patients who underwent one-stage and two-stage bilateral VATS pulmonary resections. To our knowledge, this is the largest cohort of bilateral pulmonary resection research comparing one- and two- stage surgery after bias balanced with propensity score matching method. We found that most patients in both one and two-stage group had an uneventful postoperative recovery. Among all the 151 VATS cases, there was only one intraoperative thoracotomy conversion due to lymph node adhesion. Post-operative complications were observed in 33 out of 109 one-stage surgery (30.28%) and 14 out of 42 two-stage surgery (33.33%). Our results were similar to other researches, which indicated that one-stage VATS surgery had comparable outcomes and relatively low morbidities for SBLL patients with two-stage surgery [6]. In addition, we developed a nomogram with three risk factors that related to the one-stage surgery complications. The nomogram had reasonable prediction value and could be incorporated into surgical decision making for SBLL patients.

Complications after surgery were often the major concern for surgeons and patients. Our findings may reduce the concern for these complications. Similarly, some other study also reached the same conclusion. For example, Fu reported one-stage VATS surgery for 18 bilateral pulmonary nodules and all patients recovered well without major complications [7]. Tong and his colleagues studied 16 cases of their centre who received one-stage surgery in single-utility port VATS. Minor complications including air leakage, atrial fibrillation and poor healing of the surgical site occurred [8]. Yao also reported 29 one-stage VATS included lobar-lobar resections, lobar and sub-lobar resections, and sub-lobar-sub-lobar resections. Most of the patients had uneventful postoperative course except for one severe dyspnoea. There was no significant difference in complications and chest tube days between one-stage and two-stage patients at the same time [9]. However, there were some slight differences in the recommendations on the resection scope. For example, Kohno recommended at least one side sub-lobectomy for safe reasons after studying 19 one-stage bilateral surgery patients [10], which is in accordance with Yao’s paper. Another study directly compared one-stage bilateral VATS and unilateral resection. One-stage surgery showed similar post-operative complication incidence and hospital stays and achieved a safe postoperative recovery as long as wedge resection was performed on one side [11]. Table 4 summarises the peri-operative results of previous research [2, 4, 7, 12]. Overall, our study and previous investigations all favoured one-stage approach and suggested at least one sub-lobectomy in one-stage bilateral resection.

**Table 4:** summary of previous one-stage surgery results

Researchers	Pulish date	Cases	Duration of Chest Tube	Postoperative Hospital Days	Complications	Year
Zhang et al.	2018 Oct	56	n/a	5.39 ± 2.67	8	2016–2018
Fourdrain et al.	2021 Jun	55	6.7 ± 6.2	9.2 ± 6.3	28	2011–2018
Qu et al.	2020 Apr	34	2.8 ± 3.1	4.2 ± 4.3	10	2016–2019
Xu and Fu	2019 Feb	18	5.28 ± 2.67	12.58 ± 5.87	3	2016–2018
Matsubara et al.	2018 Mar	19	2.79 ± 0.3	8.7 ± 0.9	0	2009–2016
Yao et al.	2016 Mar	29	4.0 ± 1.2	7.3 ± 1.8	9	2009–2014

One-stage VATS was proved to be an equally safe procedure with two-stage VATS, though, it was still a major procedure with around 30% of post-operative complication rate. Although not life threatening in most cases, some complications would nevertheless bring extra sufferings for the patients. In order to select the best candidates for one-stage surgery, we developed a nomogram based on the multivariate logistic regression for the risk factors to predict those who may experience post-operative complications. Moreover, efforts must be made to reduce the risk for patients even they are predicted to be “low risk” candidates. Careful preoperative evaluation, meticulous operation, and advanced post-operative management all contribute to good recovery. We noticed that patients in one-stage group had significantly more hydrothorax (40%) than those in two-stage group (11.1%). The reason might be that the coughing movement was weakened as wound pain was sever in the one-stage bilateral surgery group. Enough analgesic should be prescribed to relief the postoperative pain.

In this study, both wedge resection (WR) and anatomical segmentectomy (AS) were categorized as sub-lobectomy (SL), as they are similar in the surgical trauma. It is generally recommended that lobectomy should be performed for large, progressing, and invasive lesions, while sub-lobectomy for the small, non-invasive ones. The choice of sub-lobectomy (AS or WR) was determined by the location, size, and consolidation/tumour ratio (CTR) of the lesion. If two-stage resection is planned, lobectomy is preferred in the first stage, and an interval of 3–4 months before the next surgery is recommended. Some researchers also claimed that in the one-stage surgery, wedge resection should be performed on at least one side to reduce the surgical trauma [11]. Meanwhile, for malignant lesions diagnosed before operation, systematic lymph node dissection should be considered as long as the patients could tolerate the surgery [13]. For patients who could not tolerate or refuse surgery, and those with multifocal malignant lesions which could not be resected radically, some investigators proposed that epidermal growth factor receptor tyrosine kinase inhibitors (EGFR-TKI) treatment may be an alternative as long as the biopsy reported positive EGFR mutation [14].

Overall, one-stage VATS surgical treatment achieved favourable results for the SBLL patients, with acceptable postoperative complications. The nomogram would further help select patients with lower “risk”. However, our study is not without limitations. First, this was a retrospective study with inevitable selection bias. Future investigation in a larger cohort multicentre prospective study is required. Also, as an analysis focusing on the safety and feasibility of one-stage surgery, both benign and malignant lesion were included. As a consequence, long-term survival analysis for cancer patients was absent. In the cancer subset of the bilateral patients, it would be more powerful if prognostic results of 5-year overall survival and recurrence-free survival were presented to determine whether one- or two-stage bilateral surgery is appropriate. Last, the nomogram for the prediction of postoperative complications did not get a perfect predictive value. Large sample size is required to build a more powerful model.

## Conclusion

One-stage VATS for synchronous bilateral lung lesion patients was proved to be a safety procedure. The risk and severity of postoperative complications in one-stage VATS were comparable to the two-stage VATS, but less surgical trauma and cost, thus one-stage VATS could be a possible choice for the treatment of synchronous bilateral lung lesion patients. Advanced age, pre-surgical low haemoglobin and blood loss may predict complications after surgery. Patients with those risk factors should be paid more attention postoperatively.

## Data Availability

The datasets used and/or analysed during the current study are available from the corresponding author on reasonable request.

## Abbreviations

VATS: Video assisted thoracic surgery
SBLL: Synchronous bilateral lung lesions
PSM: Propensity score matching
LDCT: Low dose computed tomography
AUC: Area under ROC curve
ROC: Receiver operating characteristics
C-index: Harrell Consistency Index
NLR: Neutrophils/lymphocyte ratio
PLT: Platelet
HB: Haemoglobin
OR: Odds ratio
CI: Confidence interval
WR: Wedge resection
AS: Anatomical segmentectomy
SL: Sub-lobectomy
CTR: Consolidation/tumour ratio
EGFR: Epidermal growth factor receptor
TKI: Tyrosine kinase inhibitors
